# Gender role orientation and body satisfaction during adolescence – Cross-sectional results of the 2017/18 HBSC study

**DOI:** 10.25646/6901

**Published:** 2020-09-16

**Authors:** Emily Finne, Marina Schlattmann, Petra Kolip

**Affiliations:** Bielefeld University School of Public Health, Department Prevention and Health Promotion

**Keywords:** GENDER ROLES, GENDER STEREOTYPES, BODY IMAGE, BODY SATISFACTION, ADOLESCENCE

## Abstract

During adolescence both sexes experience a loss of body satisfaction, whereby the effect is greater among girls. Coming to terms with gender roles is an important step in the development of a person’s identity. Traditional gender roles tend to emphasise certain physical attributes: attractiveness in women, and strength and dominance in men. This article analyses associations between a traditional gender role orientation and body satisfaction during adolescence based on logistic regression models and using data taken from the 2017/18 Health Behaviour in School-aged Children (HBSC) study (n=1,912 girls, n=1,689 boys).

The results show an overall high degree of body satisfaction, with girls scoring lower than boys. Role preconceptions were mostly not traditional, with boys being slightly more traditional than girls. In both sexes, a more traditional role orientation was accompanied by lower levels of body satisfaction; in boys, this effect was seen to decrease with age.

The stereotypical features of role preconceptions are examined as a possible explanation for these differences. An alternative explanation posits that an egalitarian role orientation (i.e. one based on the principle of equality) creates a more tolerant environment with greater social support, which could foster a greater sense of self-acceptance.

These results indicate that questioning traditional preconceptions of gender roles during adolescence may help prevent problems related to body image in both sexes.

## 1. Introduction

Differences between the sexes during adolescence are evident in numerous indicators of physical and mental health, as well as of health behaviour [1, 2]. While during childhood the health of boys is more vulnerable, as children enter adolescence, girls more frequently report psychosomatic complaints or lower levels of well-being [3, 4].

Explanations for these differences are often generally based on a differentiation between biological sex and social gender, whereby the relevance of gender and the construction of a gender identity is highlighted [1]. A conclusive empirical explanation for these differences depends on the collection of data for social gender indicators. However, it remains unclear how to capture gender through empirical studies. Equally, there are no convincing concepts to dissolve our binary construct of sex [3]. Conceptually, it is safe to assume that social aspects of sex (gender) can be depicted at different levels, ranging from the individual to the social. International empirical surveys thereby reveal that greater equality at the societal level is related to a greater physical and mental well-being of adults and adolescents of both sexes [4–7].

To further clarify these correlations at the individual level, the current cycle of the Health Behaviour in School-aged Children (HBSC) study [8] applied an instrument to survey traditionally oriented gender role preconceptions as an element of gender. Gender roles consist of individual and socially shared stereotypical concepts of typical traits for girls/women and boys/men, and the behaviour believed typical and acceptable based on ascribed gender [9, 10]. Coming to terms with role preconceptions associated with sex is one of the central developments a person experiences during adolescence. In this phase, people go through a number of physical, mental, and social processes of maturation and these processes shape their self-image [11]. During this process, adolescents develop an understanding of the degree to which the body that they develop during puberty corresponds to society’s concepts of femininity or masculinity. Such concepts therefore gain greater personal importance [12].

From today’s perspective, expectations surrounding gender, e.g. that it is important for girls to be a good mother and wife, and that boys should develop leadership qualities and show authority, can be interpreted as corresponding to more ‘traditional’ concepts of gender roles. In Germany, only a small fraction of adolescents hold such traditional views, whereby such views are more common among boys, adolescents with a migration background and adolescents with low levels of education than among girls, adolescents without a migration background and those with a higher level of education [13–16]. Notably, since the turn of the century, attitudes towards gender roles in Germany have continued to evolve into a more egalitarian model [17].

Role preconceptions also play into expectations regarding attractiveness: according to traditional gender roles, it is more important for girls to be pretty and attractive for the opposite sex in order to find a husband, whereas for boys, being attractive has traditionally played a lesser role [10, 12, 18].

Being satisfied with one’s physical appearance and body is an aspect of one’s body image, a concept which concerns the feelings, thoughts, judgements and in some cases behaviours related to how one perceives one’s own body [19]. Body satisfaction thereby focuses on an overall assessment of a number of bodily traits and can be conceptualised as an element of subjective well-being [20, 21]. The importance of body satisfaction generally increases during adolescence [22, 23]. Lower levels of body satisfaction are related to risky health behaviour in both sexes, such as weight reduction and extreme body shaping and considered a key risk factor for eating disorders [24, 25].

Body dissatisfaction is more common among girls than boys [26, 27]. The usual explanation is that during puberty the bodies of girls do not tend to develop in line with Western ideals of slimness, while the changes boys undergo (such as muscle and beard growth) bring them closer to masculine ideals. Whereas the pressure to be ‘good-looking’ was for a long time deemed to apply almost exclusively to girls, boys and men are today observably also coming under increasing pressure to conform to socially determined body ideals. The increasing number of images of (ideal) male bodies in the media, for example, is indicative of an increasing objectification of male bodies [28, 29]. This is accompanied by increasing body dissatisfaction also among boys [19, 30, 31]. Most surveys, however, focus on dissatisfaction with weight, a problem which is more relevant to girls, and focus less on other aspects such as a muscular appearance, which is more crucial for boys [32–34].

As concepts of attractiveness are bound directly to gender [19, 35–37], there is a good case to believe that the pressure to conform to specific female and male ideals is also related to adolescents’ internalised gender roles. Some studies with adolescents indicate that in both sexes a stronger identification with typically female traits is a risk factor for problems with body image and eating disorders, whereas a greater identification with male connoted traits is a protective factor [38]. On the other hand, it appears to be the case that for both girls and boys the risk of developing eating disorders increases when self-image differs from the stereotypical gender norm, but the risk for body dissatisfaction does not [39].

With regard to body image, few surveys have so far assessed the role traditional gender norms, which create gender inequality, play with regard to body image during a phase in which reconciling a gender role with one’s self-image is an important developmental step.

More traditional gender roles could result in girls placing greater importance on being attractive and therefore potentially increase their susceptibility to problems with body image. Indeed, studies have shown that among women, traditional concepts of femininity are associated with a greater desire to be slim [34].

For boys, with regard to traditional gender roles, it can be assumed that physical appearance is not as important. Certain findings concerning adolescents appear to corroborate this fact [14, 40]. However, the traditional emphasis on physical strength and superiority of the male gender can be reflected in the expectation of having a muscular body, potentially leading to body dissatisfaction in boys with traditional role preconceptions who do not meet this ideal. Some empirical studies have shown that traditional concepts of masculinity among young men are associated with a more pronounced desire to have a muscular appearance [34, 41–43].

This article analyses how an individual’s gender role orientation, i.e. the preconceptions of typically female or male traits, privileges or gender expectations, are related to the body satisfaction of adolescent girls and boys. As both body image and also the importance an individual places on gender roles are processes in development, we also analyse how the studied relations develop with age.

## 2. Methodology

### 2.1 Sample design and study implementation

The 2017/18 HBSC study used a written questionnaire, which was handed out to students in years five, seven and nine. A multi-stage procedure was used to randomly select schools across Germany, and school classes at these schools were again randomly selected for the survey. The German data set collected data from a total of 4,347 adolescents (2,306 girls and 2,041 boys). To correct for deviations in terms of representability with regard to federal state, type of school, sex and age group, the analyses were conducted with weighted data. A detailed description of the HBSC study and its methodology are included in the article by Moor et al. [8] in this issue of the Journal of Health Monitoring.

### 2.2 Surveying instruments

Using a questionnaire, adolescents were asked about gender role preconceptions, body satisfaction, height and weight, and data was collected on indicators for family affluence and migration background, as well as month and year of birth.

Data on gender role perceptions was collected based on a shortened version of the Attitudes Toward Women Scale for Adolescents [44, 45]. On a five-point scale, adolescents were asked to state to which degree they agreed with five statements on female and male gender roles and traits. Table 1 contains the wording of these items. Values ranged from zero to four, with higher values indicating a greater approval of traditional gender role concepts.

With a Cronbach’s alpha of 0.85 for the total sample (girls=0.83; boys=0.85), the unidimensional scale achieved satisfactory internal consistency. Scale values were calculated as mean values of the item if at least four of the five items were answered.

To measure body image, this analysis used measures that are not focussed on specific body parts, which often have a very different meaning for girls and boys; instead, it looked more generally at how satisfied adolescents were with their physical appearance. Data on satisfaction with one’s appearance was collected via a sub-scale of the Body Investment Scale (BIS) [45, 46]. The scale collects data for six statements related to emotion-based attitudes to one’s body and appearance on a five-point scale (Table 1).

Cronbach’s alpha for the scale was 0.88 in the total sample (girls=0.90; boys=0.83). A mean value was calculated for items if at least five out of the six items were answered.

Data provided on body height and weight was used to calculate corresponding Body Mass Indexes (BMI), and year of birth data to calculate age at the time of the survey. Based on the study design, three age groups were differentiated: 11-, 13- and 15-year-olds, whereby the actual age of students in these age groups hovered around the median value. Family affluence was measured based on the Family Affluence Scale (FAS) [47]; a possible migration background was defined through data on the country of birth of adolescents and their parents [8].

### 2.3 Statistical methodology

Mean values and standard deviations for girls and boys were defined and compared via t-tests and/or U tests between girls and boys. Gender role and body image distribution were heavily skewed, which is why median values and interquartile ranges are also provided. All analyses were conducted with weighted data. Statistical analyses applied R software (version 3.5.1 [48]) using the packages ‘survey’ [49] and ‘ggplot2’ [50].

For the predictive models, we dichotomised body satisfaction (also due to the skewed distribution) based on the median: the lower 50% of values for girls and boys were classified as (sex-specific) low body satisfaction, the upper 50% constituted the reference group with relatively high body satisfaction. This led boys to be classified as dissatisfied if their value on the BIS scale was below 3.4, and girls as (relatively) dissatisfied if their value on the BIS scale was below 3.0.

The dichotomised values for body dissatisfaction were predicted using logistic regression models for both girls and boys. The models predict the probability for body dissatisfaction depending on how strongly a person upholds traditional gender role orientations. Yet, because the probability of body satisfaction, next to sociodemographic background variables such as age group, family affluence and migration background, also depends on further factors, the model also included Body Mass Index (BMI). Odds ratios were calculated for the models.

**Figure 1:**
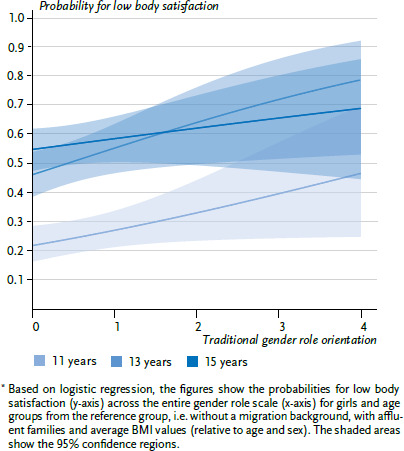
Predicted probabilities for greater body dissatisfaction among girls (n=1,912)^*^ Source: 2017/18 German HBSC study

**Figure 2:**
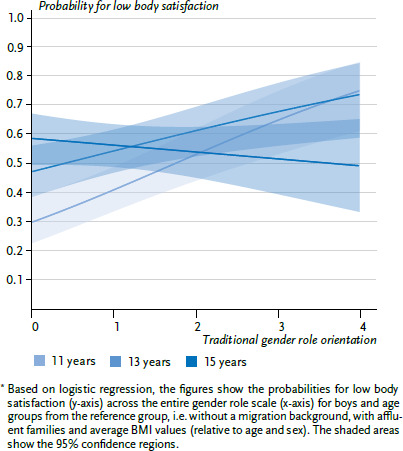
Predicted probability for greater body dissatisfaction among boys (n=1,689)^*^ Source: 2017/18 German HBSC study

In a further step, the interactions between role orientation and age group as well as BMI were tested. To illustrate interaction effects with age, the predicted associations between role orientation and the probability for low levels of body satisfaction are visualised (Figure 1 and Figure 2) for the three age groups.

## 3. Results

### 3.1 Description of the sample

Weighting was applied to achieve parity between the number of girls and boys in the sample. There was also roughly the same number of students in each of the three age groups, with a median age of 13.4 (standard deviation (SD)=1.71). Table 2 presents the values for the measures used for both sexes. Significance values refer to differences between the medians for girls and boys.

The scale for gender role preconceptions was significantly skewed. Most adolescents in the German HBSC study rejected traditionally orientated gender stereotypes, and a majority therefore achieved low values on the scale. Approval of traditional orientations was even slightly lower for girls (median=0.26) than for boys (median=1.01).

On the scale with values ranging from zero to four, a majority of adolescents revealed a high degree of body satisfaction. Body satisfaction for boys (with a median value of 3.38) was significantly higher than for girls (median=2.96).

Both for role preconceptions and body satisfaction, the differences by sex are statistically significant.

### 3.2 Predicting lower body satisfaction

The results (Table 3) for girls show that the probability of being dissatisfied with physical appearance increases significantly with age and increasing BMI. The value also increases with decreasing family affluence. No association was found with migration background.

Among female respondents, upholding traditional gender roles significantly predicted body satisfaction. Girls with more traditional preconceptions of gender roles were less satisfied with their physical appearance. An increase by one unit on the scale for traditional role orientation was associated with a 30.8% greater risk (odds) for less body satisfaction. Having a less affluent family also increased the risk for a lower level of body satisfaction. No meaningful statistical interaction between gender role and either age or BMI was found for girls (data not shown).

The odds ratio indicates the relationship between the probabilities for dissatisfaction versus satisfaction. The probability itself is easier to interpret. Figure 1 shows the probability of girls being less satisfied with their physical appearance dependent on their age and how strongly they uphold traditional role orientations. The results show an increase in the probability of greater body dissatisfaction for 11-year-olds from about 22% for those holding the least traditional views to 47% for those holding the most traditional gender role orientations. The probabilities for 13- and 15-year-old girls were correspondingly higher. For 15-year-olds, the increase in probability was slightly (but not significantly) lower.

Body dissatisfaction also increased with age in boys, although this mainly occurred between the 11- and 13-year-old age groups, where the risk (odds) increased by nearly 37%, just as it did with BMI. An increase by one BMI point translated into a 9.4% higher risk (Table 3). However, both associations are less pronounced than for girls. For boys, no meaningful association between migration background or family affluence and body dissatisfaction could be shown. Traditional gender role orientations also had a smaller effect than shown among girls, but were nonetheless statistically significant: an increase by one point in the approval of traditional role orientations increased the odds for greater dissatisfaction with physical appearance by 26.8% among boys.

Table 3 presents the main effects of the reported predictors. Further evaluations also showed a significant interaction between age and gender role among boys, and this is shown in Figure 2: with age, the correlation between traditional role orientation and greater body dissatisfaction decreases among boys. For the group of 15-year-olds, no meaningful relation is found. For a boy from the 11-year-old reference group with an average BMI (relative to age and sex), Figure 2 indicates a predicted probability for greater body dissatisfaction of about 30% for those scoring lowest on traditional role orientation, and around 75% for those with maximum scores for traditional role orientation. In contrast, among 15-year-old boys, the probability varies between 49% and 59%, whereby a more traditional role orientation is even related to a lower probability for dissatisfaction.

## 4. Discussion

During adolescence, the bodies of girls and boys undergo considerable changes. They develop sex specific adult body traits, and during this phase, therefore, preconceptions of typically female and male attributes and behaviours gain increasing personal importance. At this age, conflicts with body image become more frequent too [23]. In many cases, this goes hand in hand with risky health behaviour, i.e. diets, excessive exercise or even eating disorders [24, 25].

This analysis has looked at the degree by which traditional gender roles relate to greater body dissatisfaction among girls and boys.

### 4.1 Associations between gender role orientation and aspects of body image

Adolescents of both sexes in general showed high satisfaction with their body and physical appearance. For other frequently used measures in questionnaires, such as judging body weight, the corresponding levels of satisfaction are considerably lower. In the most recent HBSC study, for example, around half of all girls and boys believed that they were either slightly or far too fat or too thin [51]. Such an assessment of body weight, however, could be less negative than generally assumed. Therefore, adolescents may not consider their weight to be ‘perfect’, but that does not necessarily mean that they reject their physical appearance outright. Moreover, the emotionally charged and in some cases extremely negative statements (‘I hate my body’) used in the scale, in contrast to the question on weight, could provide a further explanation for the more positive results.

Similar to other measures of body image [51], body dissatisfaction was greater among girls and the data confirmed that with rising BMI, the probability of greater dissatisfaction increases, in particular for girls. Here too, there was also a clear increase in dissatisfaction with age, a finding that confirms earlier studies [23, 52], and is often explained by the increase in body fat that is a normal part of puberty for girls.

The results highlight that in both sexes traditional gender role orientations are connected to body image. The more strongly students upheld conservative preconceptions of female and male roles, the greater the probability for them to be less satisfied with their bodies. This applied independently of weight, family affluence, migration background and BMI. However, in boys, this effect decreased with age. Among 15-year-old boys, the association was no longer found and, as a matter of fact, appears to become inverse.

Our results contradict the findings of an earlier analysis, which – unlike many other studies – had asked very similar questions on gender role orientation and body satisfaction [14]. For adolescents in Berlin, the study found that greater adherence to traditional role orientations correlated to greater body satisfaction. This can presumably be explained, at least partly, by the fact that the group of those holding traditional views comprised a particularly high percentage of boys and students of both sexes at lower secondary schools (‘Hauptschule’), who expressed high body satisfaction. The study showed lower values for self-esteem for those with traditional gender orientations, which, like our results, indicates that traditional orientations are related to lower levels of subjective well-being.

The associations found by the HBSC study for girls can potentially be explained by the fact that appearing pretty is a trait traditionally ascribed to females. Girls whose self-image is shaped by this ideal and who believe that being pretty is an important facet of their identity as a woman, would accordingly be more critical if they consider that they deviate from their ideal, and this would create greater dissatisfaction. Such an interpretation is supported by analyses of young women which found that traditional concepts of femininity go hand in hand with a strong desire to be slim [34].

Ideals of masculinity, in contrast, are traditionally focused on other traits than being physically attractive. But typically male-connoted traits would be physical strength and dominance. Not having a strong, muscular body could then cause body dissatisfaction among boys that have internalised and compare themselves to these role expectations.

International research has shown that rigid preconceptions of masculinity cause problems with body image in adult men [43, 53]. A muscular body is thereby seen as a way to express masculinity [29, 41]. Young men that describe themselves as possessing typically male traits thereby less frequently reported problems with body image and eating disorder symptoms [38], whereas those that did not fit the typical male expectations had a higher risk of developing eating disorders [39].

The approach is reductive and based on uniform stereotypical ideal concepts, such as those used by the media and in advertisements that adolescents consume. More nuanced differences in preconceptions of femininity and masculinity by social background are not taken into account here. In future surveys, it would be interesting to conduct a more nuanced analysis of such differences in the ideals adolescents hold. In particular regarding the documented shift in gender roles, such research could prove insightful [17].

At this stage we can only speculate as to why the found relations become weaker in the group of elder boys. Potentially, the desire to fulfil traditional role expectations is stronger in boys of this age in other areas of their lives (such as in their attitudes to risk or sexuality). No data was collected on whether adolescents ascribe masculine traits to themselves. Body developments that come naturally with age (such as a deeper voice and beard growth) could lead boys to perceive themselves as more masculine, correspondingly leading to a decrease in the pressure to conform (or not to conform) to a masculine body ideal.

Looze et al. [4] provide an alternative explanation for the connections to gender role orientations. In an international analysis of the 2009/10 HBSC study, they found that greater gender equality in society was related to a higher satisfaction with life among adolescents of both sexes. This finding was explained empirically through higher levels of social support in countries with higher levels of equality. They concluded that greater gender equality is accompanied by greater recognition of values connoted as feminine, such as tolerance, co-operation, and social support, which would then positively affect well-being in both genders.

Based on this interpretation, less traditional role preconceptions and, related to this, a perceived greater equality between sexes, could increase the perception of tolerance, social support and personal freedom to be who you are. This could decrease the pressure to conform physically to a specific ideal and lead to a greater acceptance of one’s physical appearance. Unlike an explanation that is based on the assumption that different ideals exist for adolescent girls and boys, this interpretation would imply that less obviously perceived differences between the genders affect both sexes through the same mechanism and in an equal manner. This could be underpinned by girls and boys showing similar associations between body satisfaction and gender role orientation.

Ultimately, based on our current analysis, we can only make assumptions on the nature of the correlations found. The hypotheses regarding tolerance and social support could, however, be analysed further with current HBSC data. Whether the described values, thereby, are actually connoted as female would require further analysis.

Similar to studies that found correlations between levels of gender equality in society and diverse indicators of health and well-being [4–6, 54], we see here that traditional gender role orientations are associated with negative outcomes that are health relevant for both girls and boys. Even though the corresponding correlations do not confirm a causality, the studies mentioned do indicate that it is not only girls and women who benefit from a shift in gender roles. Almost all studies also show that for boys and men, traditional conceptions of gender and gender inequality are accompanied by diverse negative effects on health and well-being.

Thereby, the surveyed adolescents predominantly rejected traditional role conceptions. Regarding this point, no internationally comparable data from the HBSC study is currently available. However, a symmetric distribution of the scale has been reported internationally [44]. The, by contrast, skewed distribution found in Germany, with a majority of adolescents holding less traditional views, as well as Germany’s relatively high scores for gender equality in an international comparison [4, 6], allow us to conclude that traditional role conceptions are probably less pronounced among German adolescents compared, for example, to adolescents from Eastern European countries. It is therefore possible that the scale applied does not allow for adequate differentiation within the group studied. It would be interesting to conduct similar comparisons internationally.

The finding that, in absolute figures, few adolescents hold traditional views with regard to gender, and that the levels for boys are slightly higher, is confirmed by previous national studies [13–16]. The Shell study [55] is the only one to reach a different conclusion; however, this study only asked respondents how they thought employment responsibilities should be shared between a couple, and the methods of data collection are not comparable.

### 4.2 Strengths and limitations

The strengths of the HBSC study are its large representative sample from across Germany as well as its international comparability. Regarding the interpretation of the findings in this paper, it is nonetheless important to highlight some methodological shortcomings. Cross-sectional data collection does not allow causal links to be established. It is not possible to corroborate that gender role orientation has a causal effect on body image. Moreover, for some values, there are large gaps in the data, in particular for BMI. For comparisons, these analyses were therefore reran without this variable and with a greater number of cases. No meaningful differences were found in the results. As only few adolescents provided high values for traditional role orientation, estimating the probability for greater body dissatisfaction becomes imprecise at the upper end of the scale (broad confidence interval). Furthermore, the questions on a traditional division of roles that appeared in the instrument might already be irrelevant for today’s generation of adolescents in Western Europe. Future analyses should therefore adapt this instrument. A study such as HBSC, which has been designed for international comparative analyses, can invariably live up to its strengths more effectively in international comparisons. Moreover, no comparative values from large-scale studies are available, either for gender role orientation or for the applied scale on body satisfaction, that would enable a clear interpretation of what constitutes a high level of satisfaction or orientation towards traditional values. To ensure better data visualisation, body satisfaction was thus split into two groups along the gender-specific median value. The selection of cut-off values can thereby influence results. Here, the international comparative HBSC results will provide further insights. Further analyses will show how relevant the scale content is with regard to how today’s adolescents view gender roles.

### 4.3 Conclusions

Our findings indicate that internalised traditional gender roles have consequences for body satisfaction and therefore are a factor in well-being for both sexes during adolescence. Accordingly, an orientation towards classical gender roles appears to be associated with negative consequences that begin to appear even during adolescence. The survey of adolescent role orientation by the HBSC study will allow a future analysis of such interrelations, including for other indicators of health and health behaviour, as well as a further exploration of other possible explanations. Evaluations of international HBSC data allow comparisons to be made between adolescents from different societies where the influence of traditional attitudes varies widely.

From a broader perspective, the results indicate that already in adolescence, greater gender equality could serve for promoting a positive body image as an important indicator of well-being. Questioning stereotypical gender role ideals, which has been proposed as a pillar for the prevention of body image issues among girls [56], could in future also be more widely considered for boys. This supports the public health goal to further decrease gender-related health inequalities.

## Key statements

- A majority of adolescents rejected traditional gender roles; boys were slightly more traditional than girls.
- A majority of adolescents reported a high degree of body satisfaction, whereby girls were less satisfied than boys.
- In both sexes the strength of traditional role preconceptions was a significant predictor of greater body dissatisfaction.
- Only among boys, there was evidence of an age-dependent association between gender role orientation and body satisfaction.
- The results highlight the relevance of social gender for the prevention of body image problems during adolescence.

## Figures and Tables

**Table 1:** Items used to collect data on traditional gender role orientation and body satisfaction^*^ Source: Galambos et al. 1985 [44], Inchley et al. 2018 [45], Orbach & Mikulincer 1998 [46]

Shortened version of the Attitudes Toward Women Scale for Adolescents as a measure of traditional gender role orientation	Sub-scale of the Body Investment Scale (BIS) on emotional attitudes towards body and physical appearance as a measure of body satisfaction
More encouragement in a family should be given to sons than daughters to go to college.	I am frustrated with my physical appearance.
In general, the father should have greater authority than the mother in making family decisions.	I am satisfied with my physical appearance.
It is more important for boys than girls to be well at school.	I hate my body.
Boys are better leaders than girls.	I feel comfortable with my body.
Girls should be more concerned with becoming good wives and mothers than desiring a professional or business career.	I feel anger toward my body.
	I like my appearance in spite of its imperfections.

^*^ The five-point response scale ranged from ‘strongly agree’ to ‘strongly disagree’. During evaluation, the values were coded between zero and four, whereby higher values stand for a more traditional role orientation or higher body satisfaction.

**Table 2:** Distribution in the sample of traits for which data was collected (n=2,306 girls, n=2,401 boys)^*^ Source: 2017/18 German HBSC study

Girls		Boys	Total	Significance comparison between groups
**Traditional gender role preconceptions**				
Mean value (SD)	0.56 (0.70)	1.12 (0.96)	0.84 (0.88)	p<0.001
Median	0.26	1.01	0.60	
Interquartile range	0.04–0.95	0.23–1.79	0.103–1.355	
Missing (%)	3.0	4.3	3.7	
**Body satisfaction (BIS)**				
Mean value (SD)	2.81 (0.93)	3.22 (0.72)	3.01 (0.86)	p<0.001
Median	2.96	3.38	3.19	
Interquartile range	2.20–3.56	2.83–3.82	2.52–3.70	
Missing (%)	0.9	1.6	1.2	
**Body Mass Index**				
Mean value (SD)	19.34 (3.72)	19.51 (3.80)	19.42 (3.76)	p=0.164
Missing (%)	14.1	12.2	13.1	

SD = Standard deviation, BIS = Body Investment Scale (value range: 0 to 4), traditional role preconceptions = Attitudes Toward Women Scale (value range: 0 to 4), Missing = missing data

^*^ Percentage data calculated according to weighted data; absolute figures refer to frequencies in the unweighted sample.

**Table 3:** Results of binary logistic regression models to predict lower body satisfaction (n=1,912 girls, n=1,689 boys) Source: 2017/18 German HBSC study

Predictor	Girls		Boys	
	OR	(95% CI)	OR	(95% CI)
**Traditional gender role orientation**	**1.31**	**(1.12–1.53)**	**1.27**	**(1.15–1.41)**
**Age group**				
11 years (Ref.)	1.00	–	1.00	–
13 years	**2.76**	**(2.17–3.50)**	**1.37**	**(1.70–1.74)**
15 years	**3.71**	**(2.93–4.70)**	1.25	(0.96–1.62)
**Family affluence**				
High (Ref.)	1.00	–	1.00	–
Medium	1.26	(0.97–1.62)	0.91	(0.70–1.19)
Low	**1.37**	**(1.01–1.87)**	1.08	(0.76–1.55)
**Migration background**				
None (Ref.)	1.00	–	1.00	–
One-sided	1.07	(0.81–1.42)	1.02	(0.74–1.40)
Two-sided	0.98	(0.78–1.21)	1.16	(0.92–1.47)
**Body Mass Index**	**1.14**	**(1.10–1.17)**	**1.09**	**(1.06–1.13)**

OR = Odds ratio, CI = Confidence interval, Ref. = Reference group, Bold = significant effect (p < 0.05)

## References

[1] Bucksch J Finne E Glücks S (2012) Die Entwicklung von Geschlechterunterschieden im gesundheitsrelevanten Verhalten Jugendlicher von 2001 bis 2010. Gesundheitswesen 74(1):S56–S62 22836893 10.1055/s-0032-1312635

[2] Pitel L Geckova AM van Dijk JP (2010) Gender differences in adolescent health-related behaviour diminished between 1998 and 2006. Public Health 124(9):512–518 20723949 10.1016/j.puhe.2010.05.005

[3] Rommel A Pöge K Krause L (2019) Geschlecht und Gesundheit in der Gesundheitsberichterstattung des Bundes. Konzepte und neue Herausforderungen. Public Health Forum 27(2):98–102

[4] de Looze ME Huijts T Stevens GWJM (2018) The happiest kids on earth. Gender equality and adolescent life satisfaction in Europe and North America. J Youth Adolesc 47(5):1073–1085 29019054 10.1007/s10964-017-0756-7 PMC5878193

[5] Kolip P Lange C Finne E (2019) Gleichstellung der Geschlechter und Geschlechterunterschiede in der Lebenserwartung in Deutschland. Bundesgesundheitsbl 62(8):943–951 10.1007/s00103-019-02974-2 31165173

[6] Kolip P Lange C (2018) Gender inequality and the gender gap in life expectancy in the European Union. Eur J Public Health 28(5):869–872 29767703 10.1093/eurpub/cky076

[7] Viner RM Ozer EM Denny S (2012) Adolescence and the social determinants of health. Lancet 379(9826):1641–1652 22538179 10.1016/S0140-6736(12)60149-4

[8] Moor I Winter K Bilz L (2020) The 2017/18 Health Behaviour in School-aged Children (HBSC) study – Methodology of the World Health Organization’s child and adolescent health study. Journal of Health Monitoring 5(3):88–102. www.rki.de/journalhealthmonitoring-en (Stand: 16.09.2020) 10.25646/6904 PMC8734187 35146275

[9] Eckes T (2008) Geschlechterstereotype: Von Rollen, Identitäten und Vorurteilen. In: Becker R (Ed) Handbuch Frauen- und Geschlechterforschung. Theorie, Methoden, Empirie, Band 5. VS Verlag für Sozialwissenschaften, Wiesbaden, P. 171–182

[10] Ellemers N (2018) Gender Stereotypes. Annu Rev Psychol 69:275–298 28961059 10.1146/annurev-psych-122216-011719

[11] Quenzel G (2015) Entwicklungsaufgaben und Gesundheit im Jugendalter. Beltz Juventa, Weinheim

[12] Kågesten A Gibbs S Blum RW (2016) Understanding factors that shape gender attitudes in early adolescence globally: A mixed-methods systematic review. PLOS ONE 11(6):e0157805 27341206 10.1371/journal.pone.0157805 PMC4920358

[13] Demircioglu J (2017) Geschlechterrollen- und Vaterschaftskonzepte bei Jugendlichen in Deutschland. Der Pädagogische Blick 25(3):156–168

[14] Valtin R Wagner C (2004) Geschlechterrollenorientierungen und ihre Beziehungen zu Maßen der Ich-Stärke bei Jugendlichen aus Ost- und Westberlin. Z Erziehwiss 7(1):103–120

[15] Knothe H (2002) Junge Frauen und Männer zwischen Herkunftsfamilie und eigener Lebensform. In: Cornelißen W Gille M Knothe H (Eds) Junge Frauen - junge Männer. Daten zu Lebensführung und Chancengleichheit. Leske + Budrich, Opladen, P. 89–134

[16] Gille M (2006) Werte, Geschlechtsrollenorientierung und Lebensentwürfe. In: Gille M Sardei-Biermann S Gaiser W (Eds) Jugendliche und junge Erwachsene in Deutschland. Lebensverhältnisse, Werte und gesellschaftliche Beteiligung 12- bis 29-Jähriger. VS Verlag für Sozialwissenschaften, Wiesbaden, P. 131–211

[17] Röhr-Sendlmeier UM Gabrysch K Bregulla M (2018) Einstellungen zu Erziehung und Partnerschaft – ein Zeitwandel von 2009 bis 2017. Kinder- und Jugendschutz in Wissenschaft und Praxis 63(3):100–106

[18] Prentice DA Carranza E (2002) What women and men should be, shouldn’t be, are allowed to be, and don’t have to be: The contents of prescriptive gender stereotypes. Psychol Women Q 26(4):269–281

[19] Grogan S (2010) Promoting positive body image in males and females: Contemporary issues and future directions. Sex Roles 63(9-10):757–765

[20] Thompson JK Burke NL Krawczyk R (2012) Measurement of body image in adolescence and adulthood. In: Cash TF (Eds) Encyclopedia of body image and human appearance. Elsevier, Amsterdam, Waltham, Massachusetts, P. 512–520

[21] Finne E (2014) Bewegung, Körpergewicht und Aspekte des Wohlbefindens im Jugendalter. Dissertation, Universität Bielefeld

[22] Eisenberg ME Neumark-Sztainer D Paxton SJ (2006) Five-year change in body satisfaction among adolescents. J Psychosom Res 61(4):521–527 17011361 10.1016/j.jpsychores.2006.05.007

[23] Röhrig S Giel KE Schneider S (2012) „Ich bin zu dick!“ Monatsschr Kinderheilkd 160(3):267–274

[24] Stice E Shaw HE (2002) Role of body dissatisfaction in the onset and maintenance of eating pathology: A synthesis of research findings. J Psychosom Res 53(5):985–993 12445588 10.1016/s0022-3999(02)00488-9

[25] Neumark-Sztainer D Paxton SJ Hannan PJ (2006) Does body satisfaction matter? Five-year longitudinal associations between body satisfaction and health behaviors in adolescent females and males. J Adolesc Health 39(2):244–251 16857537 10.1016/j.jadohealth.2005.12.001

[26] Ricciardelli LA McCabe MP (2001) Children’s body image concerns and eating disturbance. Clin Psych Rev 21(3):325–344 10.1016/s0272-7358(99)00051-3 11288604

[27] van den Berg PA Mond J Eisenberg M (2010) The link between body dissatisfaction and self-esteem in adolescents: Similarities across gender, age, weight status, race/ethnicity, and socioeconomic status. J Adolesc Health 47(3):290–296 20708569 10.1016/j.jadohealth.2010.02.004 PMC2923488

[28] Rohlinger DA (2002) Eroticizing men: Cultural influences on advertising and male objectification. Sex Roles 46(3/4):61–74

[29] Leit RA Pope HG Gray JJ (2001) Cultural expectations of muscularity in men: The evolution of playgirl centerfolds. Int J Eat Disord 29(1):90–93 11135340 10.1002/1098-108x(200101)29:1<90::aid-eat15>3.0.co;2-f

[30] McCabe MP Ricciardelli LA (2004) Body image dissatisfaction among males across the lifespan. J Psychosom Res 56(6):675–685 15193964 10.1016/S0022-3999(03)00129-6

[31] Labre MP (2002) Adolescent boys and the muscular male body ideal. J Adolesc Health 30(4):233–242 11927235 10.1016/s1054-139x(01)00413-x

[32] Cohane GH Pope HG (2001) Body image in boys: A review of the literature. Int J Eat Disord 29(4):373–379 11285574 10.1002/eat.1033

[33] Mohnke S Warschburger P (2011) Körperunzufriedenheit bei weiblichen und männlichen Jugendlichen: Eine geschlechtervergleichende Betrachtung von Verbreitung, Prädiktoren und Folgen. Prax Kinderpsychol K 60(4):285–303 10.13109/prkk.2011.60.4.285 21614841

[34] Smolak L Murnen SK (2008) Drive for leanness: Assessment and relationship to gender, gender role and objectification. Body Image 5(3):251–260 18585105 10.1016/j.bodyim.2008.03.004

[35] Luff GM Gray JJ (2009) Complex messages regarding a thin ideal appearing in teenage girls’ magazines from 1956 to 2005. Body Image 6(2):133–136 19250889 10.1016/j.bodyim.2009.01.004

[36] Diedrichs PC (2012) Media influences on male body image. In: Cash TF (Ed) Encyclopedia of body image and human appearance. Elsevier, Amsterdam, Waltham, Massachusetts, P. 545–553

[37] Pope HG Phillips KA Olivardia R (2000) The Adonis complex. The secret crisis of male body obsession. Free Press, New York

[38] Cella S Iannaccone M Cotrufo P (2013) Influence of gender role orientation (masculinity versus femininity) on body satisfaction and eating attitudes in homosexuals, heterosexuals and transsexuals. Eat Weight Disord 18(2):115–124 23760839 10.1007/s40519-013-0017-z

[39] Lampis J Cataudella S Busonera A (2019) The moderating effect of gender role on the relationships between gender and attitudes about body and eating in a sample of Italian adolescents. Eat Weight Disord 24(1):3–11 28290118 10.1007/s40519-017-0372-2

[40] Lopez V Corona R Halfond R (2013) Effects of gender, media influences, and traditional gender role orientation on disordered eating and appearance concerns among Latino adolescents. J Adolesc 36(4):727–736 23849667 10.1016/j.adolescence.2013.05.005

[41] McCreary DR Saucier DM Courtenay WH (2005) The drive for muscularity and masculinity: Testing the associations among gender-role traits, behaviors, attitudes, and conflict. Psychol Men Masc 6(2):83–94

[42] Schwartz JP Grammas DL Sutherland RJ (2010) Masculine gender roles and differentiation: Predictors of body image and self-objectification in men. Psychol Men Masc 11(3):208–224

[43] Gattario KH Frisén A Fuller-Tyszkiewicz M (2015) How is men’s conformity to masculine norms related to their body image? Masculinity and muscularity across Western countries. Psychol Men Masc 16(3):337–347

[44] Galambos NL Petersen AC Richards M (1985) The Attitudes Toward Women Scale for Adolescents (AWSA): A study of reliability and validity. Sex Roles 13(5-6):343–356

[45] Inchley J Currie D Cosma A (Eds) (2018) Health Behaviour in School-aged Children (HBSC) Study Protocol: background, methodology and mandatory items for the 2017/18 survey. Child and Adolescent Health Research Unit (CAHRU), St Andrews

[46] Orbach I Mikulincer M (1998) The Body Investment Scale: Construction and validation of a body experience scale. Psychol Assess 10(4):415–425

[47] Torsheim T Cavallo F Levin KA (2016) Psychometric Validation of the Revised Family Affluence Scale: a Latent Variable Approach. Child Indic Res 9:771–784 27489572 10.1007/s12187-015-9339-x PMC4958120

[48] R Core Team (2018) R: A language and environment for statistical computing. R Foundation for Statistical Computing, Wien. https://www.R-project.org/ (As at 22.05.2020)

[49] Lumley T (2019) survey. Analysis of Complex Survey Samples. R package version 3.37. https://cran.r-project.org/web/packages/survey/index.html (As at 22.05.2020)

[50] Wickham H (2016) ggplot2: Elegant Graphics for Data Analysis. Springer, New York

[51] HBSC-Studienverbund Deutschland (2020) Studie Health Behaviour in School-aged Children – Faktenblatt „Körperbild und Gewichtskontrolle bei Kindern und Jugendlichen”. http://hbsc-germany.de/wp-content/uploads/2020/03/Faktenblatt_KorperbildDiatv-2018-final-05.02.2020.pdf (As at 19.03.2020)

[52] Hähne C Schmechtig N Finne E (2016) Der Umgang mit dem Körpergewicht und Körperbild im Jugendalter. In: Bilz L Sudeck G Bucksch J (Eds) Schule und Gesundheit. Ergebnisse des WHO-Jugendgesundheitssurveys “Health Behaviour in School-aged Children”. Beltz Juventa, Weinheim, Basel

[53] Olivardia R Pope HG Borowiecki JJ (2004) Biceps and body image: The relationship between muscularity and self-esteem, depression, and eating disorder symptoms. Psychol Men Masc 5(2):112–120

[54] Torsheim T Ravens-Sieberer U Hetland J (2006) Cross-national variation of gender differences in adolescent subjective health in Europe and North America. Soc Sci Med 62(4):815–827 16098649 10.1016/j.socscimed.2005.06.047

[55] Wolfert S Quenzel G (2019) Vielfalt jugendlicher Lebenswelten: Familie, Partnerschaft, Religion und Freundschaft. In: Albert M Hurrelmann K Quenzel G (Eds) 18. Shell Jugendstudie. Jugend 2019 – Eine Generation meldet sich zu Wort. Beltz, Weinheim, P. 133–161

[56] Choate L (2007) Counseling adolescent girls for body image resilience: Strategies for school counselors. Prof School Counsel 10(3):317–324

